# A Fully Automated Method for Segmenting Arteries and Quantifying Vessel Radii on Magnetic Resonance Angiography Images of Varying Projection Thickness

**DOI:** 10.3389/fnins.2020.00537

**Published:** 2020-06-16

**Authors:** Sivakami Avadiappan, Seyedmehdi Payabvash, Melanie A. Morrison, Angela Jakary, Christopher P. Hess, Janine M. Lupo

**Affiliations:** ^1^Department of Radiology and Biomedical Imaging, University of California, San Francisco, San Francisco, CA, United States; ^2^Department of Neurology, University of California, San Francisco, San Francisco, CA, United States

**Keywords:** MRA, segmentation, Frangi, vessels, radii

## Abstract

**Purpose:**

Precise quantification of cerebral arteries can help with differentiation and prognostication of cerebrovascular disease. Existing image processing and segmentation algorithms for magnetic resonance angiography (MRA) are limited to the analysis of either 2D maximum intensity projection images or the entire 3D volume. The goal of this study was to develop a fully automated, hybrid 2D-3D method for robust segmentation of arteries and accurate quantification of vessel radii using MRA at varying projection thicknesses.

**Methods:**

A novel algorithm that employs an adaptive Frangi filter for segmentation of vessels followed by estimation of vessel radii is presented. The method was evaluated on MRA datasets and corresponding manual segmentations from three healthy subjects for various projection thicknesses. In addition, the vessel metrics were computed in four additional subjects. Three synthetically generated angiographic datasets resembling brain vasculature were also evaluated under different noise levels. Dice similarity coefficient, Jaccard Index, F-score, and concordance correlation coefficient were used to measure the segmentation accuracy of manual versus automatic segmentation.

**Results:**

Our new adaptive filter rendered accurate representations of vessels, maintained accurate vessel radii, and corresponded better to manual segmentation at different projection thicknesses than prior methods. Validation with synthetic datasets under low contrast and noisy conditions revealed accurate quantification of vessels without distortions.

**Conclusion:**

We have demonstrated a method for automatic segmentation of vascular trees and the subsequent generation of a vessel radii map. This novel technique can be applied to analyze arterial structures in healthy and diseased populations and improve the characterization of vascular integrity.

## Introduction

Analysis of human intracranial arterial system is important for characterizing disease entities with primary or secondary involvement of cerebral vascular circulation such as arteriovenous malformations, central nervous system vasculitis, and post-radiation vascular changes (Fischer et al., 2005; Jellinger and Attems, 2006; Minagar et al., 2006). Accurate quantification of vascular features serves to enhance understanding of the role of cerebral vasculature in these pathophysiological conditions. Delineation of the vascular architecture can furthermore aid planning of minimally invasive neurosurgical procedures through the identification of narrow pathways that are free of passing arteries. Time-of-flight magnetic resonance angiography (TOF-MRA) and MR venography using susceptibility weighted imaging (SWI) are two techniques that are commonly used to probe the 3D spatial architecture of arteries and veins, respectively. In order to quantify pathological deviations from normal, cerebral arteries and veins must first be separated from background brain parenchyma using a vessel segmentation algorithm.

Segmentation of the brain’s vessels is challenged by complex geometry, small vessel size, and limited contrast of TOF-MRA images. Specifically, the sensitivity to detect small arterioles proximal to the capillary bed is limited in most algorithms. These arteries are typically not clearly delineated in raw MRA images due to weak signal from slow blood flow. Our group and others have recently developed sequences for the simultaneous acquisition of TOF-MRA and SWI (Du and Jin, 2008; Deistung et al., 2009; Bae et al., 2010; Bian et al., 2015). Although the benefits of these sequences include shortened scan times and the ability to jointly display arterial and venous vessels without the need for coregistration, they often come with the tradeoff of less background suppression. Hence, there is a need for a robust method to identify both small and large arteries and accurately quantify the subtle changes in arterial diameters.

Maximum intensity projection (MIP) images (Arlart et al., 1995; Johnson et al., 1998) taken through the 3D image volumes acquired using a TOF-MRA sequence provide a more informative visual display for analysis of vessels and are typically used for segmentation. MIP is a volume rendering technique for 3D data that selects the maximum voxel value along a line from the viewpoint to the plane of projection. When performed at different thicknesses, this technique can provide volumetric images of the vasculature in the form of sequential cross-sectional images. Although MIP images can clearly depict the overall shapes and paths of the blood vessels and are computationally fast, the 2D projections do not provide a good sense of depth, i.e., the spatial relationship of overlapping vessels. In addition, the presence of overlapping non-vascular structures greatly affects the visualization of small vessels with low contrast, especially at larger projection thicknesses. To overcome these issues, vessel enhancement algorithms can be first applied in order to suppress non-vascular structures and improve delineation of small blood vessels.

Vessel enhancement may be intensity based, edge based (with strong gradients), or shape based. Sato et al. (1998) implemented a line filter that enhances tubular structures in images. Frangi et al. (1998) introduced the term “vesselness” as a measurement of tubular structures by observing the ratio of eigenvalues of the Hessian matrix. Another algorithm reported by Lorigo et al. (2001) is based on a co-dimension two level set method. Aylward and Bullitt (2001) implemented an algorithm that extracts the centerline of a tubular structure by tracking vessels from a seed point. A meta-analysis of the various vessel segmentation techniques was presented by Suri et al. (2002) who reported that the multiscale vesselness using Frangi generated the best contrast between vessels and background (Chapman and Parker, 2001). The Hessian-based filter employed by this method accentuates the contrast between tubular objects and the background, thus enhancing elongated blood vessels while suppressing other anatomical features and noise. The addition of multiscale smoothing, performed using multiple runs of Gaussian filtering with different sigma values, generates a multiscale filter response at each scale that can be used to determine the likelihood that a voxel belongs to a vessel of each particular diameter. While vessel visualization is enhanced using this approach, accurate quantification of vessel radii from resulting vessel score maps remains a challenge due to underestimation and overestimation of vessel scores at the boundaries manifesting as an artificial narrowing of thick vessels and broadening of thin vessels, respectively. In order to achieve accurate quantification of vessel radii, an alternate approach is needed.

While existing methods are limited to applying vessel enhancement filters to either the original non-projected 3D images or a single MIP through the entire imaged volume (Gao et al., 2011; Hsu et al., 2017; Phellan and Forkert, 2017), there has been little investigation of the influence of projection thickness on the effectiveness of vessel segmentation. Of the few studies reported, one showed similarity between vessel radii measurements extracted from parameter-dependent MIP MRA and digital subtraction angiography derived from high contrast x-ray images (Persson et al., 2004). Another group showed that MIP images using a slab thickness of 8 mm are superior in the detection of pulmonary nodules (Kawel et al., 2009). Since radiologists routinely adjust the projection thickness within an MIP section to aid their assessment of vessel size and location, efforts to address the influence of this parameter on segmentation and subsequent vessel radii measurements will, in addition to supporting discovery research, support the integration of automated algorithms into clinical practice. These techniques will compliment traditional subjective assessment of cerebral arteries and 2D in-plane measurements with a caliper (U-King-Im et al., 2007).

To overcome the above-mentioned limitations, we introduce a novel hybrid approach which we call an adaptive Frangi technique that incorporates a Euclidean distance transform (EDT) with the Frangi filter in order to preserve accurate vessel radii information. Using this approach, we describe a robust, automatic processing pipeline for (1) accurate segmentation of arteries from MRA images for different projection thicknesses and (2) quantification of vessel radii. We apply our technique to the MRA images of three healthy volunteers and synthetic images in order to compare the automatic segmentation with manual and other commonly used segmentation methods for different projection thicknesses. Vessel metrics were then evaluated in four patients whose images were acquired with varying scan protocols.

## Materials and Methods

### Subjects and Data Acquisition

Magnetic resonance angiography images were acquired from three healthy volunteers (mean age 25 ± 2.2 years) with no known cerebrovascular disease and four patients with juvenile pilocytic astrocytomas (mean age 15 ± 1.2 years). All subjects provided informed consent as required by our institutional review board and were imaged on a 7T MRI scanner (GE Healthcare, WI, United States) with a 32-channel phased-array coil (NOVA Medical, MA, United States) using a four-echo, gradient echo sequence (Bian et al., 2015) that was previously developed to simultaneously image arteries, veins, and cerebral microbleeds (CMBs), obviating the need for image co-registration and reducing the scan time. Scan parameters were as follows: voxel size = 0.46 × 0.46 × 1 mm using an in-plane 512 × 512 matrix, FOV = 24 cm, slice thickness = 1 mm, TR = 40 ms, flip angle = 25°, and TE = 2.7, 10.5, 13.2, and 20.9 ms. Flow compensation was performed in the readout direction, and echoes were partially acquired with 65% partial Fourier sampling. A multiple overlapping thin-slab acquisition was employed with three 36 mm slabs partitioned into 1 mm thick slices with 12 slices of overlap. The 3D acquisition was accelerated in the phase direction with autocalibrating reconstruction for Cartesian imaging (ARC) using an acceleration factor of 3, resulting in a total scan time of 10.6 min. The first echo was used to create TOF-MRA images and the remaining three echoes were combined to create a composite SWI image.

Because one of the potential applications of generating vessel radii maps is to look for variations between serial scans in longitudinal studies, we scanned two volunteers twice in order to evaluate whether the vessel radii map would change between successive scans due to head orientation and slab prescription. The two scans, separated by an interval of three weeks, were used to establish the amount of variability associated with our method by comparing the resulting vessel radii distribution between the two scans.

Clinical TOF-MRA scans at lower field strengths were also obtained for two of the juvenile pilocytic astrocytoma patients in order to demonstrate the practical application of the algorithm. The imaging parameters for these two clinical scans were as follows: field strength = 1.5/3T, voxel size = 0.43 × 0.43 × 0.5 mm/0.39 × 0.39 × 0.6 mm, in-plane matrix = 512 × 512, FOV = 20/22 cm, slice thickness = 1.2/1.0 mm, TR = 23/35 ms, flip angle = 18°/20°, and TE = 3.4/3.1 ms.

### Image Pre-processing and Manual Segmentation

In order to isolate the brain, skull stripping was first performed on the MRA images using a Brain Extraction Tool (BET) that is part of the FMRIB Software Library (FSL) (Smith, 2002), The TOF-MRA images were resampled to 0.23 mm^3^ resolution using bicubic interpolation in order to have better differentiation for the vessel radii map, which was followed by intensity normalization by dividing the gray scale intensity values by the maximum gray scale value. The 2D MIP was obtained by taking the projection of the entire imaged volume along the superior–inferior direction. Vessel segmentation was performed on the 2D MIP, 3D raw volume, and six other projection thicknesses (4, 8, 16, 24, 32, and 48 mm) using MATLAB. The cerebrovascular structures were manually segmented from both the entire volume of the three volunteer datasets and the central slice for each of six different projection thicknesses by a board-certified neuroradiologist (SP). Manual segmentation was performed by overlaying a thresholded image on the original scan using MRIcron software, with 50% transparency on background. This threshold of 0.5 times the maximum gray scale value was selected empirically by the neuroradiologist during manual segmentation. The segmentations were then converted to contours of each vessel for each axial slice before confirming them on coronal and sagittal planes and making any additional corrections where necessary. These manual segmentations were treated as the ground truth to determine segmentation accuracy.

Three synthetic vessel datasets were downloaded from the publicly available Vascular Synthesizer (VascuSynth) software (Hamarneh and Jassi, 2010). These datasets contain randomly generated vessel-like structures of varying widths, bifurcations, and orientations. The generated vascular volumes were rendered with MIPs. The synthetic image was resized and eroded in order to simulate the resolution and vessel sizes encountered in TOF-MRA. Since these datasets and corresponding binary ground truth segmentations were virtually generated, no manual segmentation was required.

### MIP Vessel Segmentation

The mathematics behind the Frangi “vesselness” filter for the purpose of vessel segmentation has been reported previously (Krissian et al., 2000). Briefly, the line intensity profile of the vessels is represented as a Gaussian function with a uniform intensity along the vessel. The Hessian matrix that is obtained by taking the second partial derivative describes the local curvature along the vessel, and its cross-section and its eigenvalues indicate the degree of curvature. The Frangi filter uses the eigenvalues to estimate the likelihood of tube-like structures. To address the need for multiscale smoothing, multiple iterations of Gaussian filtering with different sigma values were performed. Although the Frangi vessel enhancement method is an excellent technique for visualization, the accuracy of radii of vessels is not preserved. Our approach takes advantage of the vessel enhancement features provided by this method while maintaining accurate vessel shape.

**FIGURE 1:**
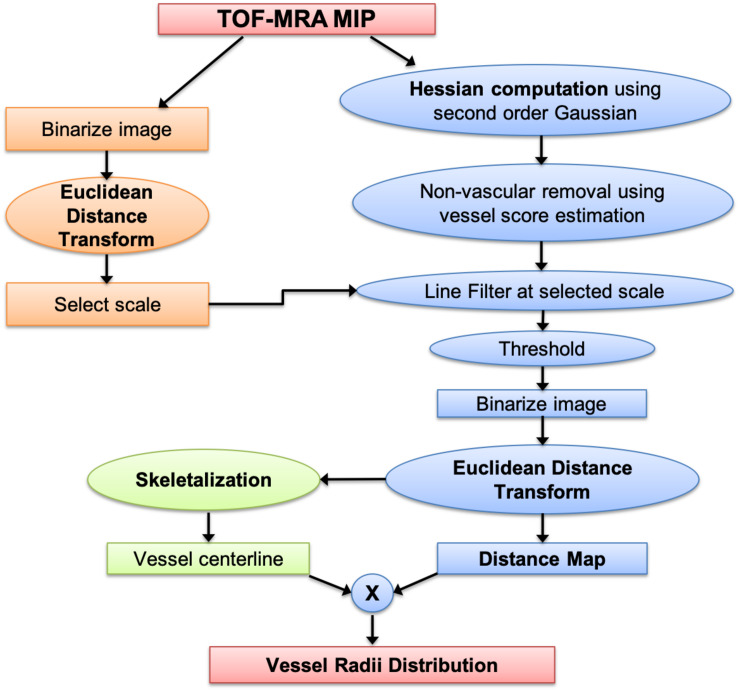
Processing pipeline for obtaining vessel radii distribution from TOF-MRA maximum intensity projection images.

**FIGURE 2:**
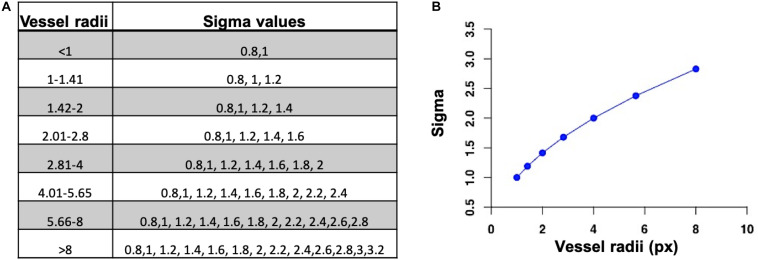
**(A)** Set of sigma values for each radii range. **(B)** Selection of maximum sigma value for the maximum vessel radii.

A flowchart of our adaptive Frangi algorithm is shown in Figure 1. This algorithm is unique because it automatically determines the appropriate set of filter scales for each vessel by first calculating the radii of large vessels using intensity-based thresholding followed by discrete distance transforms. These values are then used to determine the standard deviation values for the Frangi filter (Shikata et al., 2004). The minimum sigma value (0.8) and the increment in sigma (0.2) were chosen based on prior literature (Phellan and Forkert, 2017). The maximum sigma values were then selected based on the relation sigma_*max*_ = √ radii_*max*_ (Krissian et al., 2000). Figure 2 shows the sigma selection for each radii range. This adaptive scale selection ensures that large sigma values are included only for the detection of thick segments, and small sigma values are included only for the thin segments. From the output of the adaptive Frangi filter, fast marching (Sethian, 1996) with an intensity threshold of 0.001 was performed to obtain the binary image. A 2D EDT map, which labels each pixel of the image with the distance to the nearest boundary pixel, can then be obtained (Nystroem and Smedby, 2000). Voxel-wise measures of vessel radii were rapidly generated by employing a thinning procedure to obtain the vessel skeleton of the binary image followed by multiplication of the 2D-EDT with this skeleton to obtain the final vessel radii map. Histograms of the vessel radii map were then generated to depict vessel radii distribution in the MIP.

### Evaluation of Segmentation Performance

For the purpose of understanding the effects of projection on quantification of vessel radii, we applied our algorithm on the continuous MIPs of varying thicknesses. Our adaptive Frangi method was able to automatically determine the optimal set of sigma values for the Frangi filter for each projection. For one of the three volunteer scans, the results of automatic segmentation method were compared to the mid-slice manual segmentation done by the neuroradiologist. Dice similarity coefficients (DSC), Jaccard Index (JI), and the F-score were used as validation metrics to evaluate the segmentation agreement between the manual and automatic segmentation for each projection thickness. The DSC is a standard measure to report the segmentation performance (Dice, 1945) and measures the spatial overlap between the manual and automatic segmentation masks. The DSC is defined as twice the size of the intersection between the two masks normalized by the sum of their volumes. The DSC varies between 0 (no overlap) and 1 (complete overlap). The JI is defined as the intersection of the two binary masks divided by the union of the two masks (Jaccard, 1901). The JI is numerically more sensitive to mismatch when there is reasonably strong overlap. The F-score measures how close the predicted boundary of an object matches the ground truth boundary (Csurka et al., 2013). Both the precision and the recall of the test are used to compute the F-score: Precision is the number of correct positive results divided by the number of all positive results returned by the classifier, and recall is the number of correct positive results divided by the number of all samples that should have been identified as positive. The F-score is the harmonic average of the precision and recall, where an F-score reaches its best value at 1 (perfect precision and recall) and worst value at 0. The above-mentioned metrics are sensitive to misplacement of the segmentation label but are relatively insensitive to volumetric under- and overestimations. Hence, in addition, we determined the concordance correlation coefficient (CCC) (Lin, 1989)—a reproducibility index that evaluates the volume agreement between the manual and automatic segmented masks by measuring their combined variation from the line *y = x* (i.e., the degree through which pairs of observations fall on the 45° line through the origin). In order to compare an estimate of the segmentation error between the original and our adaptive Frangi method, we also calculated the number of segmented voxels that overlapped with the manual segmentation and divided that by the number of voxels that did not overlap for each volunteer. A paired *t*-test was then performed to test for statistically significant difference between the two methods. Repeatability of vessel radii distributions between serial scans was also evaluated for two subjects using Bland–Altman plots.

The three synthetic datasets were also used to compare the segmentation results between the original Frangi and our adaptive Frangi methods. The segmentation metrics were calculated both between the ground truth and Frangi and also the ground truth and adaptive Frangi. The number of vessel bifurcations was also calculated for the ground truth and the automatic segmentation. In order to investigate the sensitivity of our proposed method in the presence of noise, Gaussian noise with means and standard deviations of 50, 100, 150, and 200 was added column-wise to the datasets. Increasing gray scale background with a maximum of 75% of the maximum gray scale intensity value in the image was also added horizontally across the synthetic images in order to mimic different levels of background suppression of brain parenchyma. The segmentation performance was also assessed by adding noise and varying the vessel-to-background contrast of the TOF-MRA image. The DSC was calculated for the automatically segmented noise-added images with respect to the noise-free ground truth image for both the Frangi and adaptive Frangi methods. In order to evaluate the fidelity of the vessel radii estimation, the vessel radii distribution from the ground truth synthetic image was compared with the Frangi and adaptive Frangi. The same comparison could not be done for the TOF-MRA images because, with manual delineation, the vessel boundary may not be exactly drawn pixel-wise, especially for vessels with small radii, and it could not be considered as the ground truth for radii comparison.

## Results

**FIGURE 3:**
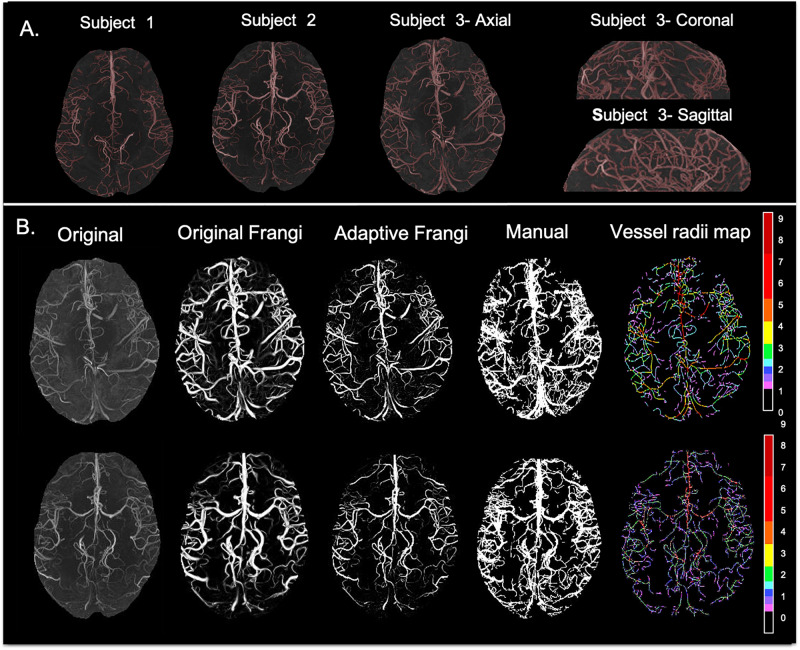
**(A)** Axial maximum intensity projection images for three volunteers along with coronal and sagittal views shown for Volunteer 3. **(B)** From left to right: original TOF-MRA maximum intensity projection; Frangi filtered image where thin vessels appear broader than expected; new Adaptive Frangi filtered image where vessels maintain the radii of the original image; manual segmentation; and color-coded vessel radii map for two volunteers. Radii values are given in terms of number of pixels (1 pixel = 0.23 mm).

Figure 3A shows the results of the adaptive filter overlaid on MRA on axial 2D MIPs for three volunteers and for one of the volunteers in the other two orthogonal views as well. The images demonstrate the versatility of our method and its performance in segmenting both small and large vessels. The Circle of Willis was not observed in subjects 1 and 3 because the 3-slab imaged volume was acquired more superiorly because we were interested in looking at smaller vessels closer to the periphery. In general, vessel diameters were decreasing toward the periphery and with increasing branching degree.

**FIGURE 4:**
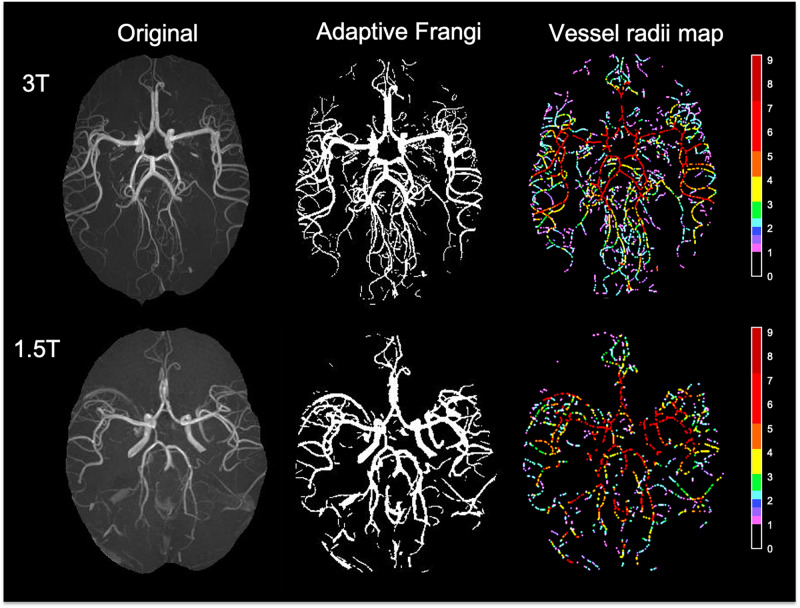
Segmentation results and color-coded vessel radii map for two clinical MRA scans of the patients with juvenile pilocytic astrocytoma acquired at 3T **(top)** and 1.5T **(bottom)**. Radii values are given in terms of number of pixels (1 pixel = 0.23 mm).

The DSC between the adaptive Frangi and manual segmentation for the three volunteers were found to be 0.89, 0.85, and 0.83, respectively. A comparison of the adaptive Frangi, original Frangi, and manual segmentation methods is shown in Figures 3B,C for two volunteers. The original Frangi filter inaccurately broadens thin vessels because it takes the maximum intensity projection across all scales, losing the thickness information of the vessels, while the manual segmentation introduces added noise after the MIP. The corresponding vessel radii distribution for the two volunteers is also shown, whereby the vessel radii map for the MIP image is color coded in terms of the number of pixels thick, where 1 pixel = 0.23 mm. Segmentation results for the two clinical MRA scans of the juvenile pilocytic astrocytoma patients acquired at lower strengths is shown in Figure 4. The repeatability test demonstrated a 2–14% percentage difference between the two serial scans for the same volunteer.

**FIGURE 5:**
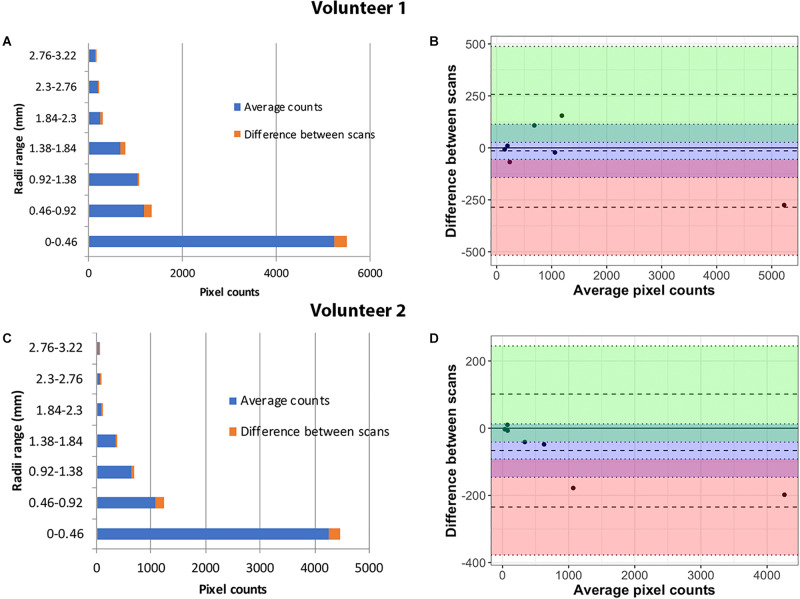
Repeatability analysis in Volunteer 1 **(top)** and Volunteer 2 **(bottom)**: Vessel radii distribution after two repeat scans **(A,C)**. The percentage variation in the counts for each radii range is marked in the chart. Bland–Altman plots show close agreement between the measurements in the two serial scans for the two volunteers **(B,D)**.

**FIGURE 6:**
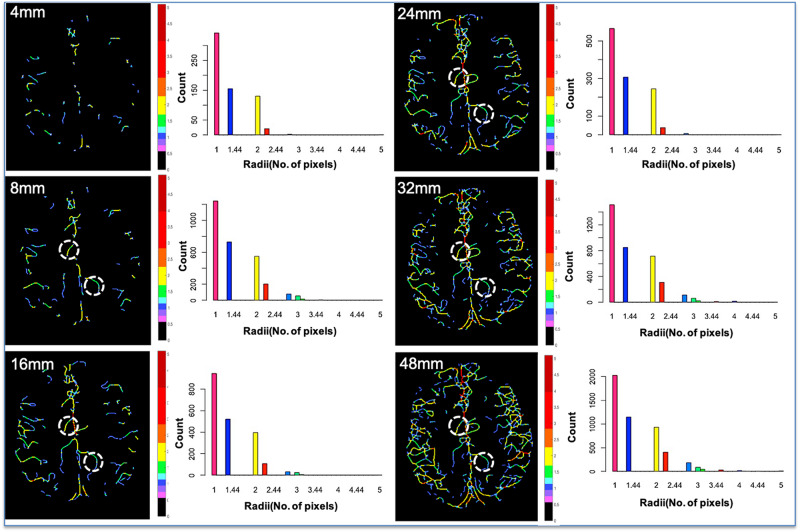
Vessel radii map for the central slice of each projection thickness and the corresponding vessel radii distribution. As the projection thickness increases, larger vessels are preserved, and the maximum radii of the vessels found in the central slice increase due to increased coverage. Although small vessels can be obscured as the projection thickness increases, the automatic segmentation from projected image improves delineation of small blood vessels. Two identical ROIs (white circles) were overlaid on each of the five projection thicknesses (8, 16, 24, 32, and 48), and the average vessel radii within that vessel segment were evaluated. The average radii measurements for the two ROIs were 1.32 ± 0.017 and 1.70 ± 0.02 pixels. The low standard deviation demonstrates the high level of precision of our method, regardless of projection thickness.

The accuracy of the method was demonstrated by means of Bland–Altman plots with lower bias, indicating that both scans were in agreement (Figure 5). Histograms of the vessel radii distribution and corresponding vessel radii map for the central slice of each projection thickness are shown in Figure 6. The maximum radii of the vessels found in a slice increased with larger projection thicknesses, likely due to increased coverage with thicker projections. Although small vessels can be obscured as the projection thickness increases, the automatic segmentation from projected image improved the delineation of small blood vessels. The average radii measurements across the five projection thicknesses (8, 16, 24, 32, and 48) for the two region of interests displayed as white circles in Figure 6 were 1.32 ± 0.017 and 1.70 ± 0.02 pixels, demonstrating the high level of precision of our method regardless of projection thickness.

**FIGURE 7:**
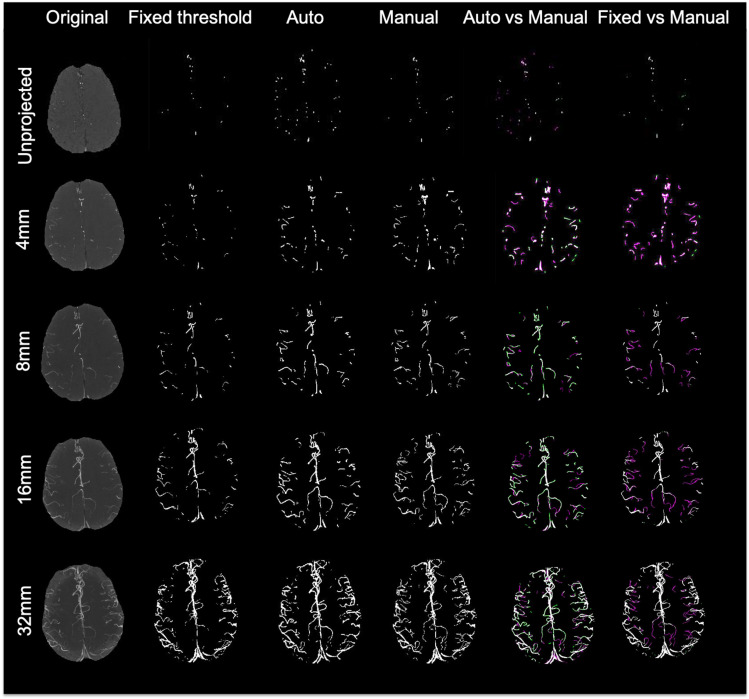
Comparison of different segmentation methods from a central slice projected at different thicknesses. For the last 2 columns, white pixels denote overlapping areas, magenta pixels depict vessels that were only segmented manually, and green pixels missed by the manual segmentation but identified by either our adaptive Frangi **(left)** or fixed thresholding **(right)**.

**FIGURE 8:**
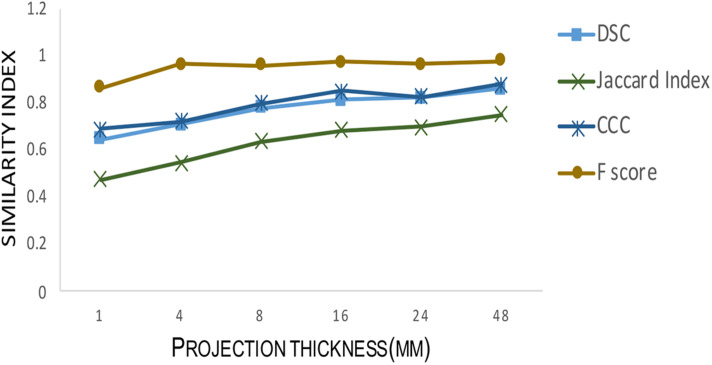
Testing segmentation accuracy between adaptive Frangi and manual segmentation for different projection thicknesses. As the projection thickness increases, the Dice similarity coefficient, Jaccard Index, F-score, and Concordance correlation coefficient between the mid-slice automatic segmentation and the corresponding manual segmentation increase.

The performance of the proposed adaptive Frangi filter was evaluated for multiple projection thicknesses (1, 4, 8, 16, and 32 mm) and compared with the corresponding manual segmentation and fixed thresholding results, as shown in Figure 7, for a representative central slice of each projection thickness. The color-coded difference images in the last two columns demonstrate that significantly more vessels were missed by the fixed thresholding method compared to the adaptive Frangi method, while the adaptive Frangi method was able to preserve vessel continuity more than either manual or fixed thresholding method. These trends persisted at all projection thicknesses, demonstrating the robustness of our method despite the increase in segmentation metrics (DSC, JI, F-score, and CCC) between the central slice automatic segmentation and the corresponding manual segmentation with larger projection thickness (Figure 8). Table 1 displays the total arterial vessel volume and the length of arteries for all the subjects based on Frangi and adaptive Frangi filtering. A statistically significant decrease in segmentation error was found with our method compared to the original Frangi (*p* < 0.003).

**TABLE 1:** Vascular metrics for nine scans.

	Arterial vessel volume (ml)		Total artery length (m)	
S.No.				
	Frangi	Adaptive	Frangi	Adaptive
1	1.20	0.80	1.91	1.78
2	1.46	1.15	2.67	2.20
3	1.08	0.76	1.73	1.53
4	1.10	0.82	2.14	1.85
5	1.86	1.23	2.72	2.34
6	1.33	1.01	2.08	1.97
7	1.15	0.98	1.96	1.88
8	1.2	0.91	1.77	1.69
9	1.1	0.89	2.25	2.11

**FIGURE 9:**
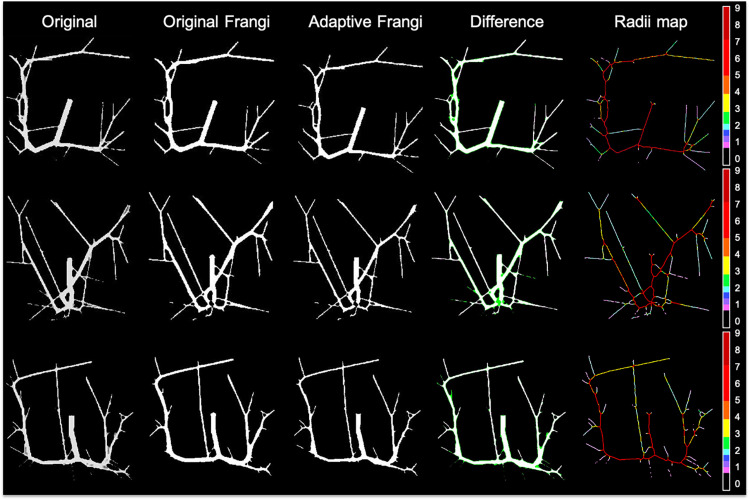
Comparison of original Frangi filtering and adaptive Frangi filtering for a synthetic dataset. In the difference image, white pixels denote overlapping areas, while green pixels represent areas missed by the adaptive Frangi filter. Both filtering strategies missed pixels at the bifurcation points.

In order to further evaluate the accuracy of our algorithm, both the Frangi and the adaptive Frangi algorithm were applied to synthetic images generated from VascuSynth software. Figure 9 shows the results of each algorithm and the difference image between the original and adaptive Frangi methods. As with the volunteer data, the adaptive Frangi method produced more accurate segmentation than the original Frangi method as shown by the minimal differences highlighted in green that only exist in cases of looped branches. The number of branches was compared in the original synthetic image and the automatically segmented image showing close agreement. The set of segmentation metrics described previously were obtained for both Frangi and adaptive Frangi compared to the ground truth synthetic image (Table 2). The DSC, JI, and F-scores were slightly higher for the adaptive Frangi compared to the Frangi method. The volume agreement metric, CCC, was also higher for the adaptive Frangi method.

**TABLE 2:** Testing segmentation accuracy in synthetic images.

	No. of branches		Dice		Dice with noise		Jaccard index		F1 score		CCC	
S.No.												
	Original	Adaptive	Frangi	Adaptive	Frangi	Adaptive	Frangi	Adaptive	Frangi	Adaptive	Frangi	Adaptive
1	38	36	0.90	0.92	0.86	0.88	0.83	0.85	0.97	0.98	0.85	0.90
2	44	40	0.90	0.90	0.83	0.86	0.82	0.83	0.99	0.99	0.90	0.91
3	56	54	0.90	0.93	0.83	0.86	0.81	0.84	0.97	0.97	0.88	0.92

*CCC, concordance correlation coefficient.*

**FIGURE 10:**
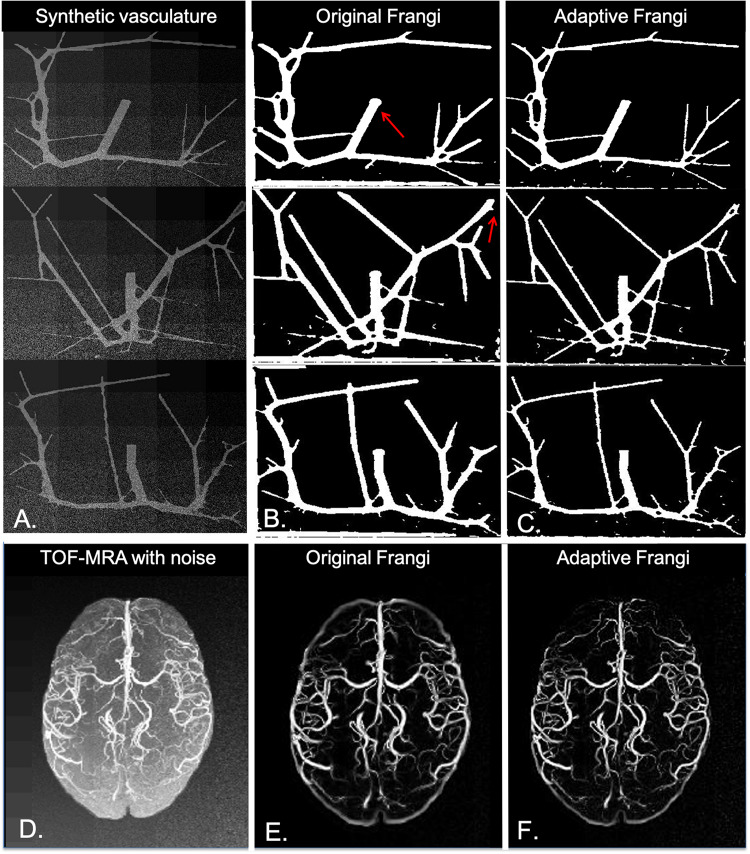
Performance of our algorithm in the presence of noise. **(A)** Noise added synthetic dataset. **(B)** Original Frangi filtered syntetic data. **(C)** Adaptive Frangi filtered synthetic data. Red arrows denote where the Frangi filter produced some bulges at the vessel tips, and two thin vessels were not clearly delineated. **(D)** Noise added to the MIP TOF image of one subject. **(E)** Original Frangi filtered MIP TOF. **(F)** Adaptive Frangi filtered MIP TOF. Adding noise reduced the DSC for this subject from 0.85 to 0.82 for the original Frangi method and from 0.89 to 0.84 for the adaptive Frangi method.

**FIGURE 11:**
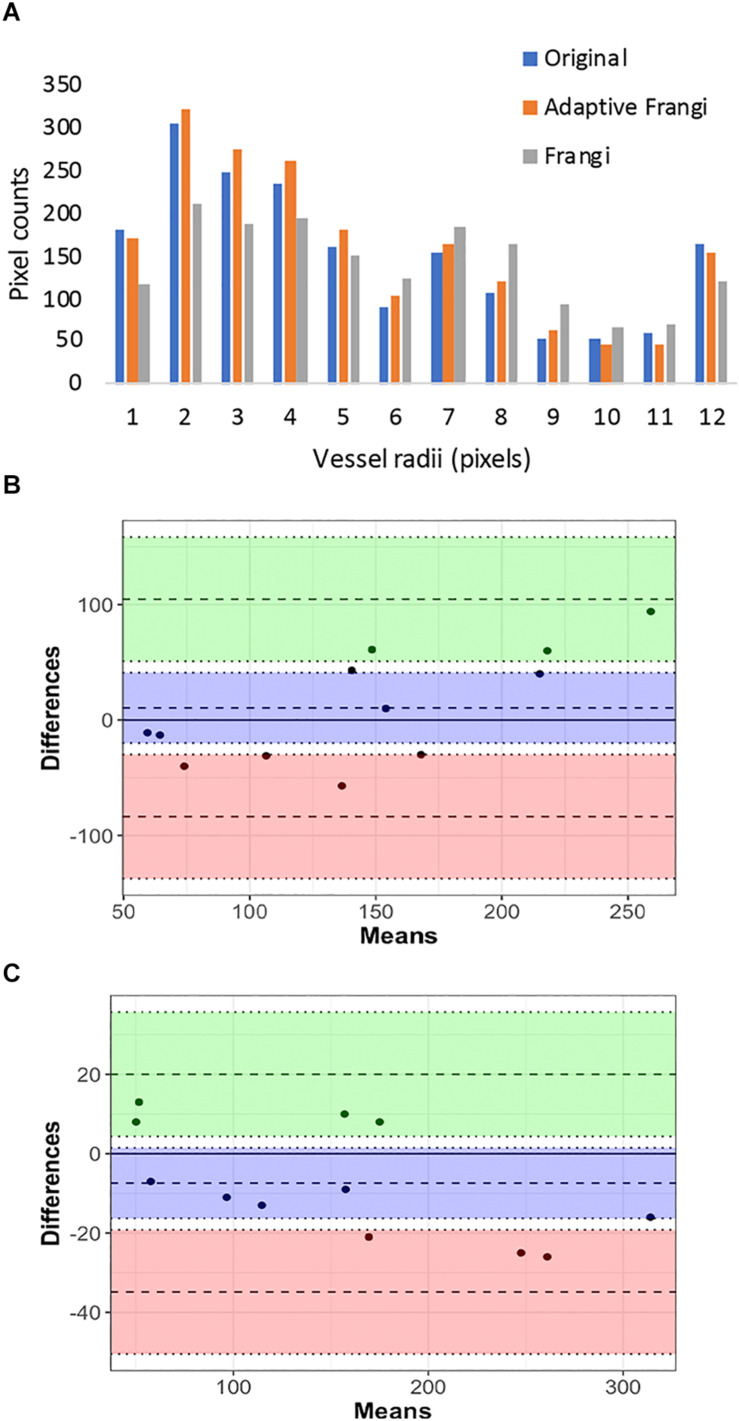
Vessel radii comparison in synthetic images: **(A)** Pixel counts for the different vessel radii obtained using the synthetic ground truth, adaptive Frangi, and Frangi. The Bland–Altman plots for the comparison of Frangi and ground truth **(B)** shows higher differences in the count compared to adaptive Frangi and ground truth **(C)**.

When tested under conditions of varying contrast and noise levels, the adaptive Frangi filter maintained accurate vessel radii throughout and outperformed the Frangi filter that erroneously produces bulges at the ends of vessels (Figure 10A). Adding noise to the TOF-MRA reduced the DSC for the subject from 0.89 to 0.82 and 0.84 for the Frangi and adaptive Frangi methods, respectively (Figure 10B). Adding noise to the synthetic images reduced the DSC as expected, with more decrease observed in Frangi compared to the adaptive Frangi method (Table 2). Regarding the fidelity of the vessel radii measured using our algorithm, Frangi and the ground truth synthetic image were compared for the three datasets and the mean count as shown in Figure 11A. The Bland–Altman plots for the comparison of Frangi and ground truth (Figure 11B) show higher differences in the count compared to adaptive Frangi and ground truth (Figure 11C).

## Discussion

Precise characterization of vascular structures is important for the assessment and objective quantification of cerebrovascular diseases. We have devised and thoroughly evaluated a new technique for automated segmentation of cerebral vascular trees from MRA images. Although the Frangi filter has been widely used in previous works, it holds limitations, and there is no standard method for determining its optimal parameters. Although Phellan and Forkert (2017) determined parameters such as minimum sigma, maximum sigma, and number of sigmas empirically, few methods (Shikata et al., 2004) have employed an EDT-based selection of scales. Our method has the advantage that it scales well for images projected at different thicknesses. The method was tested on 10 image datasets (seven from human subjects and three synthetic datasets) and validated for segmentation accuracy, flexibility, and robustness. To our knowledge, this is the first technique to explore the effects of projection thickness on the vessel filter parameters.

The TOF-MRA images used in this work were obtained from the first echo of a multi-echo sequence that simultaneously allows acquisition of SWI images (Bian et al., 2015). As a result, the parameters used for the acquisition were optimized for the combined sequence in general and not for single echo MRA images, which resulted in a longer TR than would otherwise be considered optimal. This resulted in poorer background suppression of MRA images, necessitating the use of a more powerful vessel segmentation technique. The versatility of the algorithm was then demonstrated by applying it to standard clinical MRA scans in two pediatric patients with brain tumors acquired at 1.5T and 3T and in different imaging planes. In order to make the vessel segmentation more sensitive for a given disease application, users can expand the scale range or insert intermediate sigma values in addition to the ones used in this setting.

The manual delineation of the 2D MIP and the 3D volume of continuously projected slices was performed by a neuroradiologist. We found that some smaller branching arteries were missed by our algorithm likely due to small vessels exhibiting lower contrast than larger ones (Dehkordi et al., 2011). The smoothing applied during the vessel enhancement may also contribute to this finding. Although the manual segmentation did a better job of capturing the small vessels, there were regions where our automatic segmentation visually outperformed the manual gold standard. This is because manual segmentation is prone to overestimating smaller vessels. Identification of very small, low-contrast arteries is often complicated even for expert reviewers. Manual segmentation is time-consuming as well as subject to inter-rater and intra-rater variability. In addition, the large number of arterial segments in each dataset poses a practical limit on manual segmentation by radiologists. A major issue with manual vessel diameter measurements in MRA is the variability in setting threshold levels. The estimated vessel diameter on MRA highly depends on the pixel intensity at the vessel boundaries, which is controlled by the selected image threshold (Westenberg et al., 2000).

Increasing the projection thickness has several known consequences on vessel conspicuity including the worsening of partial-volume effects, improvement of noise suppression, the preservation of larger higher-contrast vessels, and dampening of smaller vessels with lower contrast. Because increasing projection thickness improves the contrast of larger vessels, a 2D MIP is often used to increase the contrast-to-noise ratio of large vessels and accurately measure the radii of single large vessels such as the internal carotid artery or the basilar artery (Sun and Parker, 1999). Conversely, multiple non-overlapping 8 or 16 mm projections over the entire volume are more appropriate for estimating the vessel radii distribution through the entire image volume because smaller vessels with low contrast can be obscured using larger projection thicknesses. The automatic segmentation from projected images improved delineation of small blood vessels, even at larger projection thicknesses. The observed increase in similarity metrics for larger projection thickness could be due to either increased accuracy of the manual segmentations with higher projection thickness or the fact that the Frangi filter typically looks for tubular structures with higher vesselness scores compared to blob like structures. With higher projection thickness, more volume is also covered, potentially causing more vessels to appear as tubular.

Although our algorithm scales well for images of different projection thickness, it fundamentally performs assessments on 2D projection views of inherently 3D structures. This can inevitably cause loss of important vascular structure due to varying projection angles. Although other methods that consider the full 3D structure of vessels (Ilicak et al., 2016) can overcome this limitation by utilizing a reconstruction strategy that leverages vascular maps extracted from undersampled angiographic acquisitions with higher levels of background suppression, they have not been evaluated for different projection thicknesses and are far more computationally intensive, precluding the feasibility for their incorporation in clinical practice. Our method, conversely, takes only a few minutes to produce the segmented vessels on a single CPU and corresponding vessel radii maps and can be applied for any projection thickness.

Although our method can be applied to any number of images, the results for only seven human subjects and three synthetic images are presented here to discuss performance metrics in detail. The adaptive Frangi segmentation and radii estimation method should serve as a useful tool to monitor the subtle changes in arterial structure that are expected in a variety of vascular diseases. Some examples include moyamoya, atherosclerosis, radiation-induced arteriopathy, autoimmune vasculitis, and even chronic vascular disorders such as hypertension. It can aid in the automated evaluation of cerebral vasospasm after aneurysm treatment where the magnitude and pattern of vascular injury are variable in each patient (Schob et al., 2019). Another example is the evaluation of complex arteriovenous shunts, where the quantification of lesion size, location, and pattern is highly rater-dependent (Geibprasert et al., 2010). Quantification of the severity of these complex lesions using this algorithm could help to establish a much more reliable grading system as long as lumen diameters constitute at least three pixels (Hoogeveen et al., 1998).

## Conclusion

In conclusion, we have developed an automated tool for accurate segmentation of arteries from TOF-MRA images with suboptimal background suppression that provides accurate measures of vessel radii for a wide range of projection thicknesses. We have demonstrated the feasibility of applying an adaptive Frangi method on volunteer images from a 7T scanner. We believe that this approach can easily be extended to lower field strength data for routine clinical and research use given that its parameters are automatically calculated based on vessel radii, and it demonstrated superior performance on synthetic images of various contrasts and noise levels. Future work will apply this automated algorithm to study differences in vessel radii associated with normal aging, vessel pruning due to neurovascular disease, and the post-radiation angiitis.

## Data Availability Statement

The Matlab code and volunteer datasets generated for this study are available upon request to the corresponding author.

## Ethics Statement

The studies involving human participants were reviewed and approved by UCSF Institutional Review Board. Written informed consent to participate in this study was provided by the participants’ legal guardian/next of kin.

## Author Contributions

SA and JL conceived the study and wrote the manuscript. AJ performed the scans. SA and SP processed and analyzed the data. MM and CH helped with data analysis and interpretation.

## Conflict of Interest

The authors declare that the research was conducted in the absence of any commercial or financial relationships that could be construed as a potential conflict of interest.
